# The Role of Military Training in Improving Psychological Resilience and Reducing Depression Among College Freshmen

**DOI:** 10.3389/fpsyt.2021.641396

**Published:** 2021-05-17

**Authors:** Rui Guo, Meng Sun, Chi Zhang, Zebin Fan, Zhening Liu, Haojuan Tao

**Affiliations:** ^1^Department of Psychiatry, The Second Xiangya Hospital, Central South University, Changsha, China; ^2^National Clinical Research Center for Mental Disorders, Changsha, China; ^3^National Technology Institute on Mental Disorders, Changsha, China

**Keywords:** military-style training, hardiness training, college freshmen, psychological resilience, depression

## Abstract

**Background:** Military training plays an important protective role in enhancing mental health. However, the effects of military training on psychological resilience and depression among college freshmen in China remain unclear. The present study aimed to evaluate changes in psychological resilience and depression through military training among college freshmen, and to investigate associated psychosocial factors including childhood trauma that may influence its effects on psychological resilience.

**Methods:** A prospective and self-comparison study design was employed. College freshmen who received 3 weeks of military training were recruited. Socio-demographic variables were collected and childhood trauma exposure was estimated by the Childhood Trauma Questionnaire (CTQ). The Connor-Davidson Resilience Scale (CD-RISC) and Patient Health Questionnaire (PHQ-9) were used to assess psychological resilience and depression before and after the military-style training.

**Results:** The military training significantly increased the total and subscale scores of CD-RISC (*p* < 0.001), and decreased the PHQ-9 score (*p* < 0.001). The proportion of students with clinical depression reduced from 10.5% at baseline to 7.2% after the training (*p* < 0.001). Improvement of CD-RISC scores was positively affected by male gender and urban area, while negatively affected by older age, and higher baseline scores of PHQ-9 and CTQ. A significant correlation was found between changes in scores of CD-RISC and PHQ-9 through the training (*r* = −0.238, *p* < 0.001).

**Conclusions:** Military training may have a positive effect on increasing psychological resilience and reducing depressive symptoms among college freshmen, especially in male students and those from an urban area, while older age, childhood trauma, higher depression levels, and resilience at baseline may weaken, or even mask its positive effect. Follow-up research should be considered for the long-term effects of military-style training.

## Introduction

Military training, known as *junxun* in Chinese, is a combination of both theoretical teaching and physical training at schools in which students follow a soldier's daily routine and go through intensive formation training (1). Apart from basic movements such as stand at attention, stand at ease, footwork, salute, and review, some other important skills are also introduced during military training, including emergency evacuation, combat skills, self-defense, and national defense methods (2). First introduced in 1955, military training has become compulsory for all high school and university students in China since 2001. According to the Law of the People's Republic of China on National Defense Education, middle school students and undergraduates are required to have military training either before school starts in September or after National Day in October. Each year, more than 6 million college freshmen participate in mandatory military training before academic studies. Although content (physical training, cultural activities, life skills, etc.) and length of military training (2–3 weeks) may vary in different schools, their training purposes are basically identical for most students: to improve physical fitness, to enhance willpower, to develop persistence and endurance, to bear hardships, and to lead a disciplined life (1, 2). Apart from benefits for students' own growth and development, military training also promotes team spirit and team cooperation, and facilitates students' better integration and adaptation to new school life (2).

Although military training among students has sparked doubts and complaints in China, evidence from a large body of research among other populations in other countries suggests that military training plays an important protective role in enhancing resilience and reducing psychologic distress such as depression by promoting physical fitness (3–5). As the largest nonprofit institution on military health research policy in the US, the RAND Corporation has been focusing on promoting psychological resilience in the U.S. Military (3). In their recent reviews of literature, physical fitness through military training has been identified as one of the key individual factors that promote resilience (3). Similarly, several reviews and meta-analyses have also shown evidence of decreased depression associated with increased physical activities through military training (4, 5). For instance, Crowley et al. (6) examined the association between physical fitness and depressive symptoms in 300 soldiers and found a decreased risk of depression of 60% among soldiers with high physical fitness from military training. Although robust evidence has shown the benefits of military training in improving resilience and decreasing depression, little is known about its impact on the mental health of students in China.

The psychological problems of college students are an important public health issue that attracts increasing research attention (7, 8). College is a critical transitory period during which students are growing from adolescents to adults and may encounter many challenges and difficulties that render them at high risk of developing mental health problems (9). Many patients with mental disorders experienced their first psychiatric episodes while in college, and 12–18% of college students have a diagnosable mental disorder (10). Among all mental disorders reported in college campuses, depression has been one of the most frequently mentioned in previous research (11–14). For instance, a prevalence rate of 34.5% was reported for depression in one study in America (11), while another study in China reported a prevalence rate of 11.7% for depressive symptoms and 4.0% for major depressive disorder (15). It is estimated that the prevalence of depression in China is still on the rise (16). In this case, effective mental health promotion strategies to improve resilience and reduce depression among college freshmen are in urgent need.

Military training could be considered as a form of hardiness training in college freshmen. Previous studies have found that hardiness training could improve psychological resilience in the general population and contribute to mental health (17–19). Furthermore, it may have a positive impact on the prevention and treatment of depression, reducing suicide ideation, and improving the quality of life among depressed patients (17, 20). In brief, resilience-enhancing interventions including hardiness training is expected to be one of the most effective strategies of prevention and treatment of depression (17). Nevertheless, the effects of military training on psychological resilience and depression among college freshmen remain unclear. Besides, many studies suggested childhood trauma as a key risk factor of low resilience and high depression (21).

Childhood trauma refers to any physical and psychological harm suffered during childhood, which includes emotional, physical, and sexual abuse, as well as emotional and physical neglect (22, 23). Childhood trauma has been widely acknowledged as a significant risk factor for adverse mental health outcomes such as depression in later life (24). Abundant evidence has also shown that people who experienced childhood trauma have impaired resilience, and that resilience plays a mediating role between childhood trauma and depression (25). It is thus important to study the impact of military training on resilience and depression, while also taking into account childhood trauma.

To our knowledge, there are no studies that have examined the impact of military training on resilience and depression, while also considering childhood trauma in a military training sample among college freshmen in China. Given the rising trend in the incidence of mental disorders among college freshmen and the widely implemented military training programs in colleges, it is necessary to examine the effectiveness and clinical significance of military training on mental health. The purpose of this study was to evaluate the effect of military training on psychological resilience and depression among college freshmen, and to investigate associated psychosocial factors that may influence such an effect, as well as to explore the relationship between the changes of resilience and depression during the training. The findings of our study may provide valuable information and important guidance for educational management agencies in China.

## Methods

### Participants

This self-comparison design study was conducted in two universities in Changsha city of Hunan Province—Changsha College and Central South University. Our target subjects were all freshmen enlisted in military training. Eligible participants were required to be freshmen admitted to the above-mentioned two universities, who participated in the military training, and were able to read and write. Those who had serious physical or mental illnesses and thus not able to attend military training, and those who were unable to understand and communicate were excluded from our study. Finally, we recruited 8,529 college freshmen, with 2,546 from Changsha College, and 5,983 from Central South University.

### Procedures

All study procedures were approved by the medical ethics committee of the Second Xiangya Hospital, Central South University. Our research team went to each class of each university to explain the study purpose, procedures, benefits, and risk in detail. Informed written consent was obtained from all participants (or their guardians, if necessary) for the study. All eligible students were invited to fill in paper-based questionnaires both before participating in military training and after finishing the training. The surveys were distributed in small groups and self-administered with one investigator monitoring. Information on socio-demographic variables including gender, age, years of education, and hometown area (rural or urban) as well as childhood trauma were collected at baseline. Resilience and depression were assessed at 2 days prior to, as well as 2 days after the military training. All study procedures were conducted in strict accordance with the Declaration of Helsinki. All information relating to personal privacy was kept completely confidential.

### Intervention

All participants received 3-week military training, which started from 9 am to 5 pm every day including weekends. Participants were trained in small groups of 20–30 members. Each group was led by a professional instructor who was a real soldier from the army. The military instructor was in charge of the whole group and was responsible for teaching students basic movements such as stand at attention, stand at ease, footwork, salute, and review during daily training. In addition, the students also followed the lifestyle routine of the instructor, including getting up early, making up beds, making their room tidy and clean, eating food quickly, using the bathroom quickly, taking a bath quickly, obeying commands, and displaying military discipline, etc. In order to promote group cohesion and student integration, some cultural activities were also added to the daily training, such as learning military songs in a group and individual talent shows.

### Measures

#### Psychological Resilience

The Connor-Davidson Resilience scale (CD-RISC) was used to measure psychological resilience (26). This scale consists of 25 questions and each question is scored from zero to four. Consequently, the total score ranges from zero to 100, with higher scores indicating greater resilience. The CD-RISC contains five factors as follows: personal competence, trust in own intuition, positive acceptance of change, control, and spiritual influence. The CD-RISC is considered a reliable and valid instrument for measuring psychological resilience (26). In the present study, resilience was assessed by the Chinese version of CD-RISC (27) which has demonstrated adequate psychometric properties and could be a reliable and valid measurement for evaluating resilience with Chinese people (27).

#### Depression

The Patient Health Questionnaire-9 (PHQ-9) was used to assess depression before and after the training. This self-administered screening instrument (28) consists of nine items, which parallel each of the Diagnostic and Statistical Manual of Mental Disorders, fourth edition, text revision (DSM-IV-TR)-defined symptoms of major depressive disorder. To be consistent with the DSM-IV-TR major depressive disorder criteria, each of the nine depression items are rated from 0 (not at all) to 3 (nearly every day) according to symptoms in the past 2 weeks. The total scores range from 0 to 27, with higher scores indicating more severe depressive symptoms. The PHQ-9 has previously showed good internal consistency and test-retest reliability (29). In this study, we used the score of 10 as a cut-off value to estimate the prevalence of clinical depression (29, 30).

#### Childhood Trauma

The history of childhood trauma was evaluated by the Childhood Trauma Questionnaire (CTQ) (31). Content validity and reliability of the CTQ have been well-demonstrated and can be used in different populations throughout the world (32–34). It is recommended for use among adults as well as adolescents, and assesses all five types of childhood maltreatment, including emotional, physical, and sexual abuse, and emotional and physical neglect. The scale consists of 28 items, 25 of which are used to measure the five maltreatment constructs (five items for each subscale), and the other three items are used to detect cases with minimization and denial of childhood problems. In our study, history of childhood trauma was evaluated by the Chinese version of CTQ, which was demonstrated to be a reliable and valid measurement of childhood trauma in Chinese people (35). Participants were determined to have experienced childhood trauma according to the following cut-off points: ≥13 for emotional abuse; ≥10 for physical abuse; ≥8 for sexual abuse; ≥15 for emotional neglect; and ≥10 for physical neglect. Scores above these levels were considered indicative of the presence of childhood trauma. Participants were further divided into two groups—those who had experienced at least one type of abuse or neglect were designated as “traumatized,” and those who had not experienced any were designated as “not traumatized.”

### Data Analyses

All statistical analysis was performed with IBM SPSS Statistics version 20.0. Descriptive statistics were provided for socio-demographic data. Comparisons between the scores before and after the training were made for the CD-RISC and PHQ-9 through the paired *t*-test. And then the whole sample was divided into the traumatized and not traumatized groups according to the CTQ scores and the depressed and non-depressed groups according to the PHQ-9 scores at baseline. Changes in scores of the CD-RISC and PHQ-9 through the training were compared within and between different subgroups through ANOVA for repeated measurement (different genders, different traumatized status, and different clinical depression status). The proportion of students with clinical depression before and after the training were compared through the chi-square test.

To investigate the predictors of improvement in resilience through the training, we conducted multivariate linear regression analyses to calculate unstandardized coefficients (B), 95% confidence intervals for B (95% CI), and standardized coefficients (β). We entered age, gender, years of education, hometown, and scores of the CD-RISC, PHQ-9, and CTQ at baseline as independent variables, with changes in scores of the CD-RISC before and after the training as dependent variable. Correlation analysis was used to assess the relationship between changes in scores of the CD-RISC and PHQ-9 before and after the training through Spearman's correlation coefficient. We considered a *p* < 0.05 to be statistically significant.

## Results

### Description of the Sample

From the initial sample, 1,620 were excluded from analysis, with 416 refusing to participate in the study, and 1,204 failing to complete the questionnaires. A total of 6,909 valid responses were obtained. The mean age of the sample was 18.34 ± 0.92 years old, ranging from 16 to 24. More than half of the students were male (63.8%) and from rural areas (50.3%). The total years of education were 12.21 ± 0.64.

### Comparisons Between Scores of CD-RISC and PHQ-9 Before and After Military Training

After the training, the total and each subscale scores of the CD-RISC were significantly improved (*t* = −25.89, *p* < 0.001), while the average scores of the PHQ-9 significantly decreased (*t* = 24.54, *p* < 0.001) (Table 1). Additionally, the number of students with clinical depression decreased from 725 to 497 (χ^2^ = 46.67, *p* < 0.001) (Table 2).

**Table 1:** Comparisons of scores of the CD-RISC and PHQ-9 before and after military-style training.

	**Before training**	**After training**	***t***	***p***
CD-RISC				
Total score	72.93 ± 11.83	76.85 ± 13.81	−25.89	<0.001
Personal competence	24.73 ± 4.56	26.03 ± 4.63	−23.73	<0.001
Trust in own intuition	19.19 ± 3.89	23.38 ± 6.32	−52.07	<0.001
Positive acceptance of change	15.30 ± 2.78	16.10 ± 3.21	−21.33	<0.001
Control	8.39 ± 2.10	9.01 ± 2.12	−23.51	<0.001
Spiritual influences	5.32 ± 1.87	5.57 ± 1.54	−10.40	<0.001
PHQ-9	5.43 ± 3.65	4.16 ± 3.64	24.54	<0.001

*CD-RISC, the Connor-Davidson Resilience Scale; PHQ-9, Patient Health Questionnaire-9*.

**Table 2:** Comparisons of changes in scores of the CD-RISC and PHQ-9 in different groups through military-style training.

**Variables**	**Gender**			**Trauma**			**Depression**		
	**Male (*N =* 4,406)**	**Female (*N =* 2,503)**	***P***	**Yes (*N =* 4,500)**	**No (*N =* 2,409)**	***P***	**Yes (*N =* 725)**	**No (*N =* 6,184)**	***P***
Total CD-RISC^a^	4.10 ± 12.67^*^	3.50 ± 12.07^*^	0.054	2.96 ± 13.26^*^	4.37 ± 11.37^*^	<0.001	1.96 ± 13.00^*^	4.10 ± 12.37^*^	<0.001
Personal competence^a^	1.39 ± 4.60^*^	1.14 ± 4.45^*^	0.027	1.28 ± 5.04^*^	1.31 ± 4.26^*^	0.792	0.91 ± 4.67^*^	1.34 ± 4.53^*^	0.016
Trust in own intuition^a^	3.89 ± 6.46^*^	4.70 ± 7.02^*^	<0.001	3.59 ± 7.15^*^	4.50 ± 6.39^*^	<0.001	4.45 ± 6.92^*^	4.15 ± 6.65^*^	0.265
Acceptance of Change^a^	0.83 ± 2.94^*^	0.74 ± 3.37^*^	0.246	0.74 ± 3.20^*^	0.82 ± 3.05^*^	0.286	0.47 ± 3.50^*^	0.83 ± 3.05^*^	0.003
Control^a^	0.70 ± 2.23^*^	0.48 ± 2.12^*^	<0.001	0.46 ± 2.37^*^	0.71 ± 2.09^*^	<0.001	0.55 ± 2.13^*^	0.63 ± 2.20^*^	0.352
Spiritual influence^a^	0.25 ± 2.03^*^	0.20 ± 1.66^*^	0.107	0.06 ± 1.91^*^	0.36 ± 2.09^*^	<0.001	0.10 ± 1.84^*^	0.30 ± 2.05^*^	<0.001
PHQ-9^a^	−1.18 ± 4.00^*^	−1.08 ± 4.05^*^	0.098	−1.26 ± 4.89^*^	−1.14 ± 3.43^*^	0.249	−5.39 ± 4.94^*^	−0.69 ± 3.56^*^	<0.001

*CD-RISC, the Connor-Davidson Resilience Scale; PHQ-9, the Patient Health Questionnaire-9; CTQ, Childhood Trauma Questionnaire*.

a *Differences of post-training scores relative to the baseline (before training)*.

* *p < 0.001 in comparisons between post-training scores and baseline scores within each group*.

### Comparisons of Changes in Scores of CD-RISC and PHQ-9 Between Different Groups

No significant differences were found between male and female students in total scores of the CD-RISC, while the improvement in scores of the CD-RISC was much more pronounced in those without childhood trauma. No significant differences were observed in changes of scores of the PHQ-9 across genders or between the traumatized and non-traumatized groups. Participants who were not clinically depressed at baseline had greater changes in scores of CD-RISC and PHQ-9 through the training. Furthermore, changes in total and each subscale scores of the CD-RISC and PHQ-9 were all significant within each subgroup (Table 2).

### Factors Associated With Changes in Scores of CD-RISC Through the Training

No potential multicollinearity of all variables was found, with variance inflation factor values of 1.154 and below. The model was found significant (*p* < 0.001). In the multiple linear regression analysis, male gender and urban area were found to be positive factors while older age, and higher scores of the PHQ-9, CD-RISC, and CTQ at baseline played negative roles. This model accounted for 39.0% of the variance in the improvement of the CD-RISC total scores through military-style training. The CD-RISC scores at baseline were the most powerful predictors for the changes in resilience through the training (Table 3).

**Table 3:** Independent predictors of changes in scores of CD-RISC through military-style training.

**Predictor variable**	**df (CD-RISC)**				
	**B**	**95%CI**	**β**	***t***	***p***
Gender	0.601	(0.027, 1.176)	0.023	2.051	0.040
Age	−0.866	(−1.182, −0.550)	−0.064	−5.365	0.000
Education	0.454	(0.010, 0.899)	0.023	2.003	0.050
Hometown	1.427	(0.870, 1.983)	0.057	5.029	0.000
Baseline PHQ-9	−0.482	(−0.561, −0.402)	−0.141	−11.886	0.000
Baseline CD-RISC	−0.421	(−0.445, −0.0.397)	−0.400	−33.891	0.000
Baseline CTQ	−0.046	(−0.068, −0.024)	−0.046	−4.110	0.000

*CTQ, Childhood Trauma Questionnaire; PHQ-9, Patient Health Questionnaire; CD-RISC, the Connor-Davidson Resilience Scale; df, differences of post-training scores relative to the baseline (before training)*.

**Figure 1:**
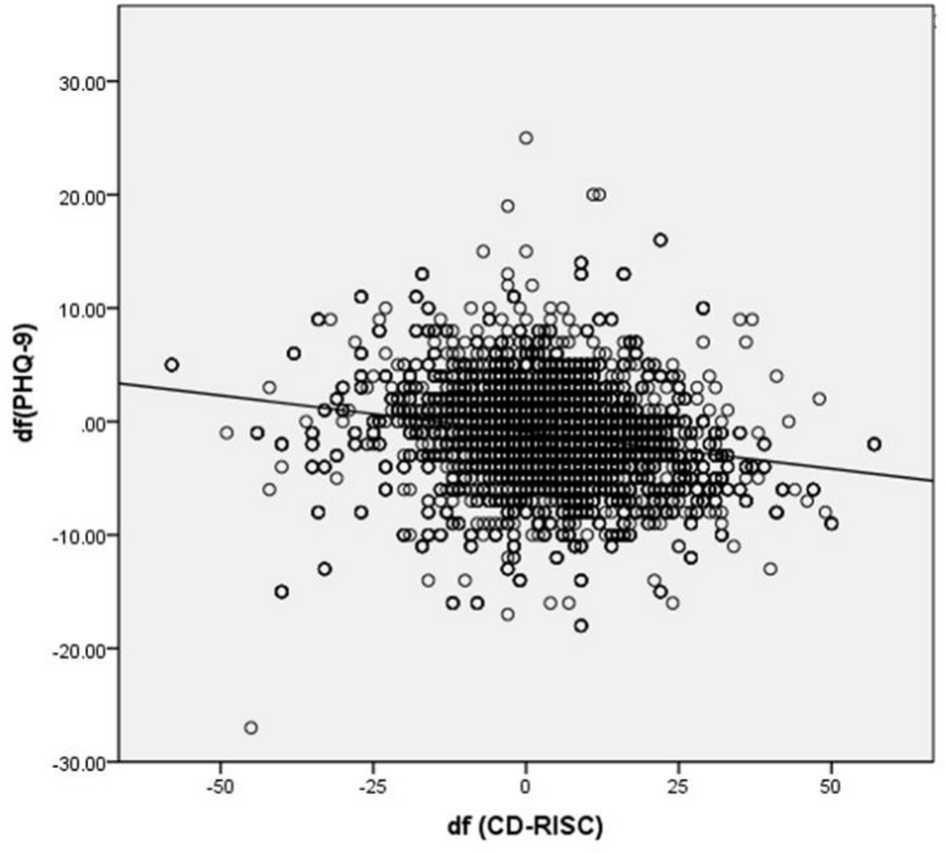
Correlation between changes scores of CD-RISC and PHQ-9 through training (*r* = −0.238, *p* < 0.001). CD-RISC, the Connor-Davidson Resilience Scale; PHQ-9, the Patient Health Questionnaire-9; df, differences of post-training scores relative to baseline scores (before training).

Changes in scores of the CD-RISC were significantly associated with changes in scores of the PHQ-9 through the training (*r* = −0.238, *p* < 0.001) (Figure 1).

## Discussion

Military training has been conducted in colleges and universities in China for over 30 years. Although the training has been proven to improve physical strength and to discipline behaviors (36), most of its benefits to mental health have been theoretical. The results of this study supported our hypothesis that military training may effectively increase psychological resilience and mitigate depressive symptoms independent of gender, trauma status, or status of clinical depression, although there are different levels of effectiveness in different subgroups.

Our main finding was that military training was effective in enhancing psychological resilience and decreasing depression. This finding adds further support to the previous reviews and meta-analyses showing the significant positive effects of physical fitness through military training on improving resilience and decreasing depression (3–5). Through military training, students develop good and healthy lifestyles such as sleeping early and getting up early, eating healthy food, and exercising intensively (37). These healthy lifestyles all help students maintain physical fitness and wellbeing, which contribute to better resilience and lower depression. In addition, one core element of military training was team building, where students may gain peer support from each other, which is also a well-known protective factor for resilience and depression (38). This finding is also consistent with a recent review on interventions to build resilience among young people (39). Among a range of interventions listed, school-based exercise programs such as military training has been proven to show significant positive effects in either enhancing resilience or preventing mental health problems (39). The study results provide support for military training as a beneficial aspect of resilience intervention and should be continued for college freshmen to improve their mental health. Another implication is that future intervention programs targeted at improving resilience and decreasing depression among college students and other populations may also consider adding and adapting some elements from military training.

Compared with students without clinical depression at baseline, those with clinical depression had less of an improvement in resilience and a smaller decrease in depression. In logistic regression, a higher level of baseline depression was also found to be a negative factor in resilience improvement. As we all know, severe depression is hard to cure without antidepressant drugs (40). Military training, as a kind of psychotherapy, may only play a limited role in improving severe depression. Students with severe depression may show a lower degree of coordination in military training, as well as less of an involvement in activities and interpersonal interaction. All of these factors will not be conducive to the establishment of a good interpersonal relationship, and thus affects the improvement of resilience. This finding indicates the necessity and importance of combining pharmaceutical treatment with military training for students with severe depression to improve their resilience.

Resilience is defined by the American Psychological Association as the process of adapting well in the face of adversity, trauma, tragedy, threats, or even significant sources of threat, and is often used to evaluate individual's social adaptive capacity and mental health (41). Previous research has found the importance of childhood rearing in resilience building (42). In our study, childhood trauma was further found to pose an obstacle to resilience despite military-style training, which suggests its long-term negative effect on mental health. In addition, although the severity of childhood trauma is correlated with the severity of depressive symptoms (43, 44), no significant differences were found in changes of depression between the two groups, which may indicate different mechanisms of improving resilience and reducing depression. Further studies are needed to investigate the associations among military training, childhood trauma, resilience, and depression.

Male gender was found to be a positive factor in improving resilience, while a critical *p*-value was found in the comparison of changes in scores of the CD-RISC, which may be caused by other confounding factors. Male students tended to be more active and to establish better relationships in the training, which may explain the positive impact. However, further research is needed to examine the long-term effect and the potential mechanism. Urban area seemed to be a protective factor, which may be explained by better adaptive ability and more familiarity with the environment. However, older age seemed to be a risk factor, which may come from more pressure and stability of the formed personality.

Another important finding is the association between resilience and depression. Alleviation of the depressive symptoms along with increased resilience through training may suggest an anti-depressive effect of resilience, which has been found in previous research (17). However, we can only partly attribute the decrease of depressive symptoms to the improvement of resilience as the changes of resilience and depressive symptoms were inconsistent in different subgroups, and the correlation coefficient between them was only 0.238. Therefore, there should be other ways to reduce depression through the training, apart from improving resilience. The development of depression has been linked to a stress-diathesis hypothesis (33), so increasing hardiness and decreasing perceived stress levels may be another way to reduce depression according to previous studies on hardness training (28).

Our study has several limitations. One major limitation is the pre-post study design which measured outcomes before and after an intervention in the same group of subjects. Without a comparison group, conclusions of this study design were solely based on the temporal relationship of the measurements to the interventions (45). Such a design may be biased since other changes occurred at the same time, or simply just the natural changes of time, may also cause changes in the outcomes, instead of the intervention (45). As a result, the observed improvements in resilience and depression in the current study may not be reliably attributed to the intervention alone, making this a weaker design than a randomized controlled study design (RCT) that uses a comparison group to control for all potential confounders. However, since military training is mandated by law in almost all higher education institutions in China, it is difficult to set up a control group who did not participate in the training. In addition, such a pre-post study design is not uncommon in public health research (46–48), we believe our study still provides useful information to guide future studies. Another solution may be using a stepped randomized controlled study design where one group received military training, while the other group delayed their training until after the study ends. Secondly, while it is promising that military training can have an immediate positive impact on resilience and depression in our study, it is unknown how long the effect can last. Long-term follow-up will be required. Thirdly, although the military training for college freshmen is similar throughout China, the training duration and the specific course arrangement may differ in different schools. So it can be argued that our results may not be available in other parts of the country. Finally, all the questionnaires were self-reported without further interview, which may weaken the accuracy of the results.

In conclusion, the present results have important implications for mental health and education promotion in colleges. Military training might play an important role in improving resilience and reduce depressive symptoms among college students. This finding provides support for military training as a beneficial aspect of resilience and depression interventions and should be continued for college freshmen. Military training also shows a potential to be adapted in other intervention programs targeted to improve resilience and mental health in other populations. Intervention effect was positively affected by male gender and urban area, while negatively affected by older age, childhood trauma, higher depression levels, and resilience at baseline. These findings suggest the need of taking into account all of these factors while developing and evaluating military training for college students, with alternative interventions provided to students with specific needs such as medication for those with severe depression. Besides, long-term effects remain uncertain, which warrants further research efforts.

## Data Availability Statement

The raw data supporting the conclusions of this article will be made available by the authors, without undue reservation.

## Ethics Statement

The studies involving human participants were reviewed and approved by The medical ethics committee of the Second Xiangya Hospital, Central South University, China. Written informed consent to participate in this study was provided by the participants' legal guardian/next of kin.

## Author Contributions

RG, MS, and ZF: data collection. RG, MS, and HT: methodology. RG: writing (original draft preparation). RG, MS, CZ, ZF, ZL, and HT: writing (review and editing). HT and ZL: project administration. All authors contributed to the article and approved the submitted version.

## Conflict of Interest

The authors declare that the research was conducted in the absence of any commercial or financial relationships that could be construed as a potential conflict of interest.
